# Homologous Delta-12 Fatty Acid Desaturase (*FAD2*) Genes Affect Gene Expression and Linoleic Acid Levels in *Lentinula edodes* under Heat Stress

**DOI:** 10.3390/jof10070496

**Published:** 2024-07-18

**Authors:** Huanling Yang, Jun Jiang, Mingjie Chen, Xiaoxia Song, Changxia Yu, Hongyu Chen, Yan Zhao

**Affiliations:** 1 Institute of Edible Fungi, Shanghai Academy of Agricultural Sciences, Shanghai 201403, China; yanghuanling@saas.sh.cn (H.Y.); mjfungi@126.com (M.C.); sxx8866@163.com (X.S.); hychen@saas.sh.cn (H.C.); 2 Lishui Institute of Agriculture and Forestry Sciences, Lishui 323000, China; jiangj-1@163.com

**Keywords:** *Lentinula edodes*, delta-12 fatty acid desaturase, differential gene expression, heat stress, bioinformatic analysis

## Abstract

Delta-12 fatty acid desaturases (FAD2s) actively regulate stress responses and cell differentiation in living organisms. In this study, six homologous *FAD2* genes were identified based on the genome sequence of *Lentinula edodes*. Then, the six FAD2 protein sequences were analyzed using bioinformatics tools, including ExPASy ProtParam, SignalP, TMHMM, and TargetP. These analyses were performed to predict the physical and chemical properties, signal peptides, and transmembrane and conserved domains of these proteins. The polypeptide sequences were aligned, and a maximum likelihood phylogenetic tree was constructed using MEGA 7.0 software to elucidate the phylogenetic relationships between homologous *FAD2* sequences. The results demonstrated that the FAD2 proteins contained three conserved histidine-rich regions (HXXXH, HXXHH, and HXXHH), which included eight histidine residues. The linoleic acid content and FAD2 enzyme activity were further analyzed, and the levels in the mutagenic heat-tolerant strain 18N44 were lower than those in the wild-type strain 18. Interestingly, the expression levels of the *FAD2-2* and *FAD2-3* genes under heat stress in strain 18N44 were lower than those in strain 18. These findings indicated that *FAD2-2* and *FAD2-3* may play major roles in the synthesis of linoleic acid during heat stress.

## 1. Introduction

Unsaturated fatty acids (UFAs), which are structural components of cell membrane phospholipids and signal transduction molecules, play a vital physiological role in the stress response of living organisms. Microorganisms acclimate to environmental factors such as nutrient supply, temperature, pH, humidity, pressure, and ionic strength by maintaining a proper proportion of liquid crystal lipids and cell membrane balance to prevent membrane injury and destruction. The mechanism includes altering the fatty acid composition of membrane phospholipids by changing the degree of fatty acid unsaturation and decreasing the lengths of fatty acid chains [1,2]. Heat stress increases the fluidity of the cytoplasmic membrane [3,4]. The degree of unsaturation of fatty acids in cell membrane lipids is widely accepted as a determinant of membrane fluidity and has previously been reported in animals, plants, bacteria, and fungi [5,6,7,8,9,10,11]. Fatty acid desaturases, key enzymes in UFA biosynthesis, are nearly ubiquitous in living organisms [12,13] and are the core regulators of many physiological reactions. Their function is to insert double bonds into the alkyl chain after removing two hydrogen atoms [14]. The first step in the synthesis of UFAs is the formation of linoleic acid. FAD2 converts oleate (18:1) to linoleate (18:2), and the expression of the FAD2 protein is temperature dependent. Research on FAD2 is particularly important for the regulation of the heat stress response in living organisms [15,16].

The basidiomycete *Lentinula edodes* (Berkeley) Pegler, known as a famous edible mushroom (shiitake), is the most economically important cultivated mushroom in East Asia and the second most popular mushroom throughout the world [17]. *L. edodes* is a type of mushroom that fruits at low temperature, and fruiting bodies of the shiitake mushroom are produced at 8–20 °C. However, the temperature of the main mushroom-producing areas in China during summer is much higher than 25 °C, causing the mycelia to become yellow or even stop growing. The agronomic characteristics of the fruiting bodies become poor (small fruiting bodies, thin cap, and less open umbrellas), seriously affecting fresh mushroom production during summer. The fluidity of the membrane is positively correlated with the unsaturation of fatty acids. Under the stimulation of rising temperature, the expression of the *FAD* gene, which transforms saturated fatty acids (SFAs) into UFAs, decreases to increase lipid saturation, thereby reducing membrane fluidity and maintaining it in the optimal state for normal biological function [18,19,20]. The tolerance of organisms to high temperature is proportional to the content of SFAs and inversely proportional to the content of UFAs. In bacteria, when *Lactobacillus coryniformis* Si3 was fermented at 42 °C for 6 h, the degree of membrane fatty acid saturation was significantly reduced, indicating that the thermal tolerance of lactic acid bacteria is closely linked with the synthesis of membrane fatty acids [21]. In fungi, fatty acids are related to temperature-induced stress and the formation of fruiting bodies in *L. edodes* [22]. In preliminary work, we evaluated the nutritional value of *L. edodes* mycelia and determined the compositions and contents of fatty acids and amino acids [23]. In this study, the fatty acid content changes in the mycelia of *L. edodes* strains 18 and 18N44 were determined by GC-MS after growth at 37 °C for different durations (0, 4, 8, 12, 18, and 24 h). The results showed that a negative correlation exists between the proportion of UFAs in *L. edodes* and its heat tolerance under high temperature, which provides a theoretical basis for the breeding of new varieties of *L. edodes* with better high-temperature tolerance [24]. In addition, the role of *FAD2* in the synthesis of linoleic acid was analyzed. We investigated the bioinformatic characteristics, elucidated the phylogenetic relationships between the homologous sequences of the *FAD2* gene and the corresponding proteins in *L. edodes*, and examined the effects of *FAD2* gene expression on linoleic acid production under different heat stresses.

## 2. Materials and Methods

### 2.1. Strains

*L. edodes* strain 18 is an off-season cultivated variety, and strain 18N44 is obtained from strain 18 by UV mutagenesis [25,26]. Our research group identified two strains (strain 18 and strain 18N44) by ISSR [27,28] and identified 18N44 as a new strain. In addition, 18N44 has the characteristics of rapid recovery and early emergence of fruiting bodies after being subjected to high-temperature stress during cultivation. In our previous studies, we determined the temperature and duration of high-temperature stress treatment [29]. *L. edodes* strain 18 and the mutagenic heat-tolerant strain 18N44 were obtained from the Institute of Edible Fungi, Shanghai Academy of Agricultural Sciences.

### 2.2. Acquisition of Gene Sequences

Six homologous genes of *FAD2* were found and downloaded from the NCBI genome database of *L. edodes* strain W1-26 (NCBI LDAT00000000, SRS875031, and SRS1090734) or the genome database of *L. edodes* (http://legdb.chenlianfu.com/; accessed on 5 January 2018) [30] and were named *FAD2-2*, *FAD2-3*, *FAD2-4*, *FAD2-5*, *FAD2-6*, and *FAD2-8*. The six homologous genes were translated into amino acid sequences by BLAST for bioinformatic analysis.

### 2.3. Bioinformatic Analysis

The protein sequences of homologous *FAD2* genes were analyzed by the following online tools. Multiple sequence alignment of the *FAD2* loci was performed with T-Coffee (https://www.ebi.ac.uk/jdispatcher/msa/tcoffee; accessed on 20 May 2018) [31] under the default settings. Protein properties were then predicted by bioinformatics tools based on analysis of the FAD2 sequences. Amino acid and atom compositions, molecular weights, and theoretical pI values were estimated by ExPASy ProtParam (http://web.expasy.org/protparam/; accessed on 20 May 2018) [32]. SignalP 4.1 (http://www.cbs.dtu.dk/services/SignalP 4.1; accessed on 20 May 2018) [33,34], TMHMM 2.0 (https://services.healthtech.dtu.dk/services/TMHMM-2.0/; accessed on 20 May 2018) [35], and TargetP 2.0 (https://services.healthtech.dtu.dk/services/TargetP-2.0/; accessed on 20 May 2018) [36,37] were used to predict signal peptides, transmembrane helices, and subcellular localization, respectively [38].

### 2.4. Heat Stress Treatment

Researchers have shown that the effects of abiotic stress on the mycelia of *L. edodes* are minimal at a temperature of 25 °C [29]. For heat stress, mycelia of strains 18 and 18N44 were inoculated on the surface of solid potato dextrose agar (PDA) plates with a diameter of 9 cm and cultured in the dark for two weeks at 25 °C. Plates covered with mycelia were placed in a blender with culture solution, and 10 mL of the resulting solution was transferred to a shaking flask (250 mL flasks containing 100 mL of potato dextrose broth (PDB) medium (Becton, Dickinson and Company Sparks, Franklin Lakes, NJ, USA), which was then incubated in a shaker at 150 rpm and 25 °C in darkness for two weeks. When the amount of mycelia in the shaking flask was sufficient, the heat stress test was applied. *L*. *edodes* mycelia incubated in liquid PDB medium were subjected to heat stress by growth at 37 °C for 4, 8, 12, 18, or 24 h; mycelia continuously maintained at 25 °C served as controls. Biological duplicates of the heat stress test were performed three times. Following heat stress treatment, mycelia in the incubation buffer were quickly collected and rinsed with sterile water under sterile conditions, flash-frozen in liquid nitrogen and stored at −80 °C for RNA extraction. All treatments were repeated in at least three independent replicates.

### 2.5. RNA Extraction and cDNA Reverse Transcription

The total RNA of the treated *L*. *edodes* mycelia was extracted using a Redzol reagent kit (Tiangen Biotech Co., Ltd., Beijing, China) according to the manufacturer’s instructions. RNA integrity was validated by agarose gel (1.5%, *w*/*v*) electrophoresis. A NanoDrop 2000 Spectrophotometer (Thermo Scientific, Rockford, IL, USA) was used to evaluate the quantity and quality of the RNA. RNA extracts with A260/280 absorption ratios in the range of 1.8–2.2 and A260/230 absorption ratios over 1.8 were selected and subsequently subjected to cDNA synthesis.

### 2.6. RT-qPCR Analysis of Gene Expression

RT-qPCR was performed in a StepOnePlus Real-Time PCR instrument (Applied Biosystems, Foster City, CA, USA) using a SYBR^®^ Premix Ex Taq™ II (Takara Biomedical Technology, Dalian, China) kit. The reaction system included 10 μL of SYBR^®^ Premix Ex Taq™ II (2×), 2 μL of template cDNA, 0.4 μL of ROX dye, 0.4 μL of each primer, and 6.8 μL of RNase-free water. The following procedures were used for PCR amplification: denaturation at 95 °C for 30 s, 40 cycles of PCR at 95 °C for 5 s, 60 °C for 15 s, and 72 °C for 15 s, and a melting curve at 95 °C for 15 min, 60 °C for 30 s, and 95 °C for 15 min. According to the 2^−∆∆Ct^ method, the relative expression of genes was measured [39,40]. The expression stability of the candidate genes of *L. edodes* mycelia under heat stress was evaluated using three native statistical software programs: geNorm, NormFinder, and BestKeeper. Our laboratory screened 10 candidate reference genes and found that beta-tubulin (*TUB*) was the most suitable internal reference gene for *L. edodes* mycelia under heat stress [41,42]. The qPCR primers that were used were designed with Primer-BLAST (https://www.ncbi.nlm.nih.gov/tools/primer-blast/; accessed on 5 May 2018) and synthesized by Sangon Biotech Co., Ltd. (Shanghai, China). The sequences of the primers used are shown in Table 1.

### 2.7. Detection of Fatty Acid Desaturase (FAD2) by an ELISA Kit

The fatty acid desaturase (FAD2) enzyme activity was measured on ice using an ELISA kit (Jiangsu Jingmei Biotechnology Co., Ltd., Yancheng, China) according to the manufacturer’s instructions. The mycelia grown on agar media were collected, and 1 g of mycelia was weighed, rinsed with PBS (pH of 7.4), and then rapidly frozen with liquid nitrogen. The sample was homogenized in PBS with a homogenizer on ice. The homogenates were then centrifuged for 20 min at 2000 rpm before the supernatant was removed. The standards or samples were added to the appropriate wells. Then, the enzyme conjugate was added to the well, which was covered with an adhesive strip and incubated at 37 °C for 60 min. The plate was then washed four times with wash buffer. After the final wash, the plates were inverted and blotted dry onto paper towels until no moisture appeared. For coloring, chromogen solutions A and B were added to each well, gently mixed, and incubated in the dark at 37 °C for 15 min. Stop solution was then added to each well. The color in the well was observed by reading the optical density at 450 nm using a microtiter plate reader. Four replicates were performed for each test.

### 2.8. Analysis of the Fatty Acid Content by Gas Chromatography-Mass Spectrometry

The linoleic acid contents in strain 18 and the mutagenic heat-tolerant strain 18N44 under heat stress were analyzed by GC-MS. The heat stress treatments of strains 18 and 18N44 were performed as described in Section 2.4. After heat stress, the samples were prepared according to the methods described by Yu et al. [23]. Briefly, mycelia (0.2 g) were acidified with 1.0 mL of 5% H_2_SO_4_, and 5 μL of nonadecanoic acid methyl ester (as an internal standard) in a screw-cap tube. The samples were then heated at 80 °C for 90 min every 10 s to eliminate N_2_ outgassing, followed by refrigeration at 4 °C for 10 min. After transferring the samples to glass vials, 0.5 mL of water and 1.0 mL of n-hexane were added, and the samples were oscillated for 20 s and then centrifuged at 2000 rpm for 10 min. Next, the supernatants were subjected to GC-MS analysis (Auto-System XL GC and TurboMass MS; Perkin Elmer, Waltham, MA, USA).

A 60 m HP-5MS capillary column with an inner diameter (i.d.) of 0.25 mm was used (Agilent Technologies, Santa Clara, CA, USA). The GC-MS instrument was programmed to begin at 70 °C for 5 min, followed by a 10 min temperature ramp up to 270 °C at a flow rate of 1 mL/min. Three replicates were performed for each sample. The samples were quantified against the internal standard, and their linoleic acid contents were expressed as the percentage of total fatty acids (FAs) present in each sample.

### 2.9. Statistical Analysis

The data were obtained from three independent replicates. Unless indicated otherwise, the data are presented as the mean ± standard error of the mean. Student’s *t* test was used for the statistical analyses. The histogram was drawn with GraphPad Prism 6 (GraphPad Software, Inc., La Jolla, CA, USA).

## 3. Results

### 3.1. Sequence Alignment and Phylogenetic Profile of the FAD2 Protein

**Figure 1:**
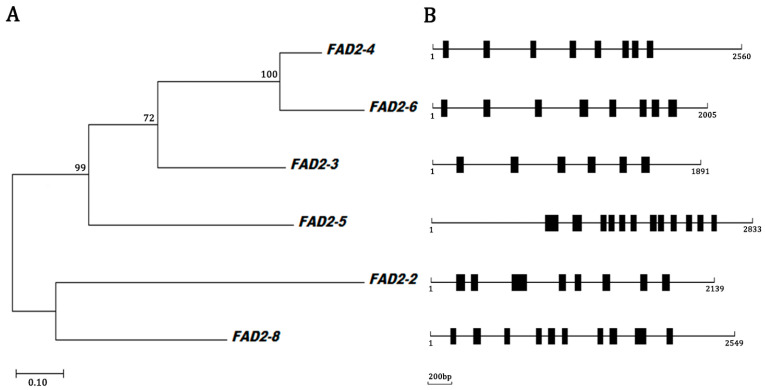
(**A**). Maximum likelihood tree of FAD2 amino acid sequences: the scale bar indicates evolutionary distance; bootstrap values from 1000 replicates are shown digitally above the branch. Sequences were aligned by the MUSCLE method, and a phylogenetic tree was constructed using Mega version 7.0. The “delete completely” option was checked to eliminate sites with coverage below 100%. (**B**). The distribution of intron locations of *FAD2* genes in *L. edodes* mycelia. The introns are shown as boxes, and the horizontal line indicates the exons of the *FAD2* genes.

To elucidate the phylogenetic relationships of the *FAD2* genes, six protein sequences were aligned. The genes were divided into two large clusters in a maximum likelihood phylogenetic tree (Figure 1A). Among these sequences, the *FAD2-3*, *FAD2-4*, *FAD2-5*, and *FAD2-6* sequences from *L. edodes* mycelia grouped into one large cluster, and *FAD2-6* and *FAD2-4* belonged to the same clade with a bootstrap value of 100%. The *FAD2-2* and *FAD2-8* sequences were grouped into the second cluster. Figure 1B shows the relative positional distribution of the *FAD2* intron in *L. edodes* mycelia. The positions of the *FAD2-5* introns are quite different from those of the other *FAD2* genes. *FAD2-2*, *FAD2-4*, and *FAD2-6* each had 8 introns. Meanwhile, *FAD2-3* contained 6 introns, which was the lowest among all the *FAD2* genes. The distribution of introns was basically consistent with the results of phylogenetic tree classification.

**Figure 2:**
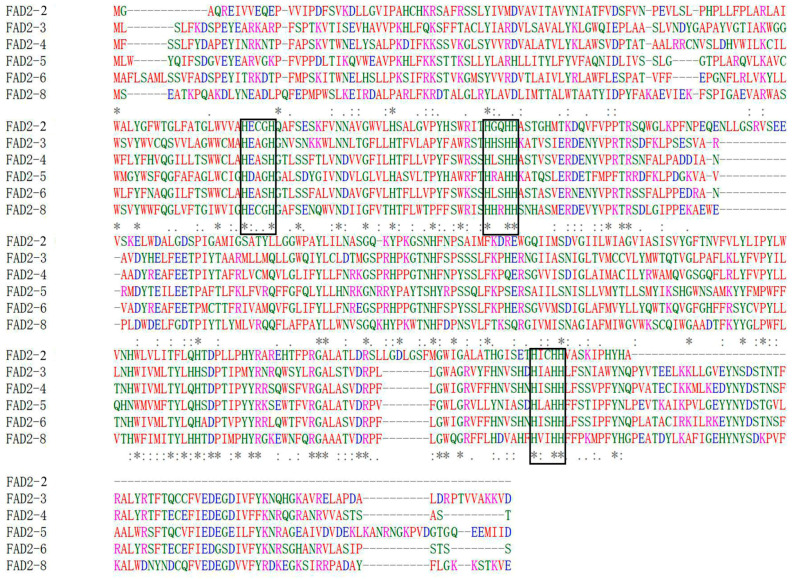
Multiple alignment of homologous FAD2 amino acid sequences from *L. edodes*. The localizations of the three histidine motifs are specified by the boxes. “*” represented conserved residues.

The close alignment of six homologous protein sequences from *L. edodes* was examined using the T-Coffee program (https://www.ebi.ac.uk/jdispatcher/msa/tcoffee; accessed on 20 May 2018). T-Coffee is a multiple sequence alignment program, and its main feature is combining results obtained using several alignment methods. The results showed that these protein sequences were strongly conserved (Figure 2). The alignment revealed that all the FAD2 polypeptide sequences contained strongly conserved histidine motifs (black box in Figure 2). The first histidine motif was HXXXH, and the second histidine motif was HXXHH. The second motif is highly conserved in the FAD2 sequence and repeats toward the carboxyl terminus of each sequence. The third motif consisted of three histidine residues. These three histidine motifs are the main functional regions involved in FAD2 activity. Sakai et al. [22] deduced that the Le-FAD2 protein has three typical histidine clusters (HXXXH, HXXHH, and HXXHH), and the catalytic domain of the Le-FAD2 enzyme is conserved in *L. edodes*, which was consistent with our analysis. The distance between the first and second motifs was relatively short (approximately 31 residues), while the distance between the second and third motifs was relatively long (approximately 202 residues), which is similar to findings in other species.

### 3.2. Bioinformatic Analysis of Homologous FAD2 Proteins in L. edodes

The ExPASy ProtParam online analysis tool was used to predict the basic physical and chemical properties of homologous FAD2 proteins from *L. edodes*. The number of amino acid residues, molecular formula, and isoelectric point of each protein are shown in Table 2. Based on the amino acid composition and the instability index, FAD2-2, FAD2-3, FAD2-5, and FAD2-8 were classified as stable proteins, while FAD2-4 and FAD2-6 were classified as unstable proteins. The proteins encoded by FAD2-2 and FAD2-6 are hydrophobic. FAD2-2 and FAD2-8 are acidic proteins, which is consistent with their predicted isoelectric points.

**Figure 3:**
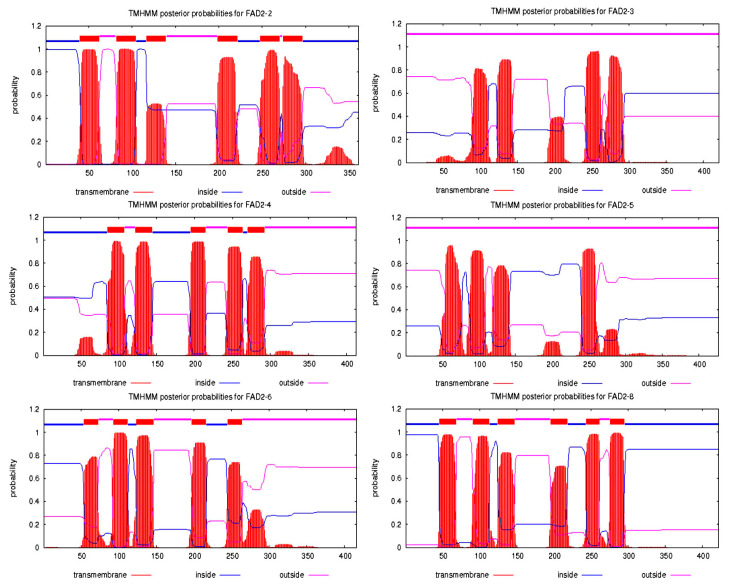
Prediction of transmembrane helices in homologous FAD2 proteins from *L. edodes*. The TMHMM server 2.0 was used to predict transmembrane helices in these proteins. Putative transmembrane domains are indicated by red boxes, and the inner and outer membranes of the cell are indicated by blue and pink lines, respectively.

The two algorithms, TargetP (http://www.cbs.dtu.dk/services/TargetP/; accessed on 20 May 2018) and SignalP 4.1 (http://www.cbs.dtu.dk/services/SignalP/; accessed on 20 May 2018), were used to predict protein sorting signals in the N-terminal region of the FAD2 sequences and the cellular localization sites of these proteins. Based on these results, FAD2-3 was the only protein located in the mitochondrial pathway, and FAD2-6 was the only protein located in the secretory pathway (Table S1). Transmembrane helices were predicted in homologous FAD2 proteins from *L. edodes* using TMHMM 2.0 (https://services.healthtech.dtu.dk/services/TMHMM-2.0/; accessed on 20 May 2018). The numbers of transmembrane domains among homologous FAD2 proteins are different, and the results are shown in Figure 3. FAD2-4 and FAD2-6 were confirmed to have five transmembrane domains, while six transmembrane helices were predicted in FAD2-2 and FAD2-8. No transmembrane helices were present in FAD2-3 or FAD2-5.

### 3.3. Differential Expression of FAD2 Genes under Heat Stress

**Figure 4:**
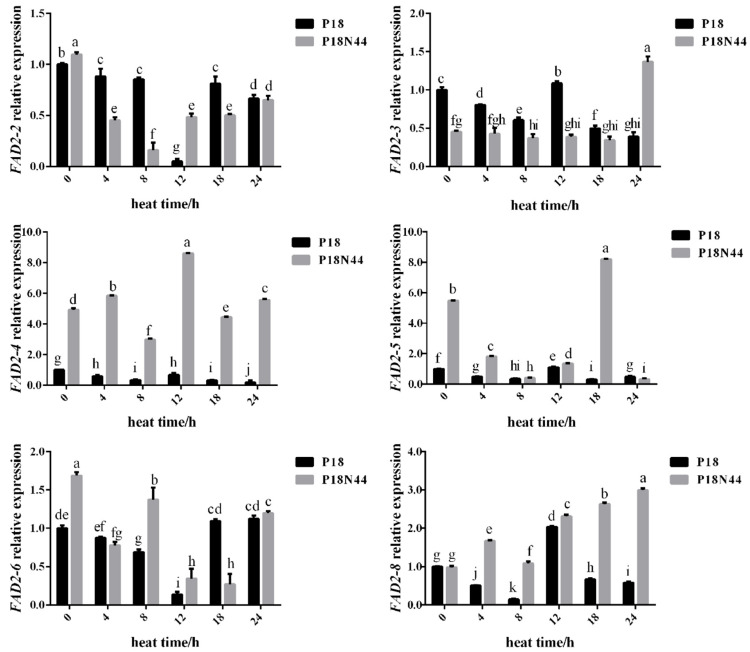
Differential expression of homologous *FAD2* genes in *L. edodes* strains 18 and 18N44 treated at 37 °C for different durations. Control (0 h) mycelia were not exposed to heat stress. The heat stress-treated (4, 8, 12, 18, and 24 h) mycelia were incubated at 37 °C. The data are the means of four independent replicates. The statistical analysis was performed using Student’s *t* test, and GraphPad Prism 6 was used to construct the histogram. Bars with different lowercase letters are significantly different at the level of 0.05 (*p* < 0.05).

The transcriptional responses of six homologous *FAD2* genes to heat stress were tested by incubating the mycelia of *L. edodes* under heat stress at 37 °C for 4, 8, 12, 18, and 24 h; mycelia that had been incubated at 25 °C were used as the control group. After the 18 and 18N44 strains were exposed to heat stress for different durations, RNA was extracted, and reverse transcription and RT-qPCR were subsequently performed to analyze the transcript levels of the *FAD2* genes. The heat-treated mycelia of strain 18 generally had lower levels of these transcripts than the unheated control samples (Figure 4). However, the heat-treated mycelia of strain 18N44 showed different trends in the expression levels of the six homologous *FAD2* genes compared with those of the unheated control samples. After heat stress, *FAD2-2*, *FAD2-5* (except for 18 h), and *FAD2-6* genes had lower transcript levels than those in the unheated control samples, and the transcript levels of all of these genes first decreased and then increased. The transcript level of *FAD2-3* decreased slightly compared with that of the unheated control sample during heat stress, whereas the expression level was significantly greater than that of the control at 24 h (*p* < 0.05). In addition, the transcript level of the *FAD2-8* gene was greater than that in the unheated control sample, which continued to increase with prolonged heat stress. Meanwhile, the transcript level of *FAD2-4* fluctuated greatly during heat stress and could be greater or less than that in the unheated control sample. The FAD2-4, *FAD2-5* (except for 24 h), *FAD2-6* (except for 4 h and 18 h), and FAD2-8 transcript levels were greater in strain 18N44 than in strain 18, but the *FAD2-2* (except for 0 h and 12 h, and no significant difference at 24 h) and FAD2-3 (except for 24 h) transcript levels were lower in strain 18N44.

### 3.4. Activity of FAD2 under Heat Stress

**Figure 5:**
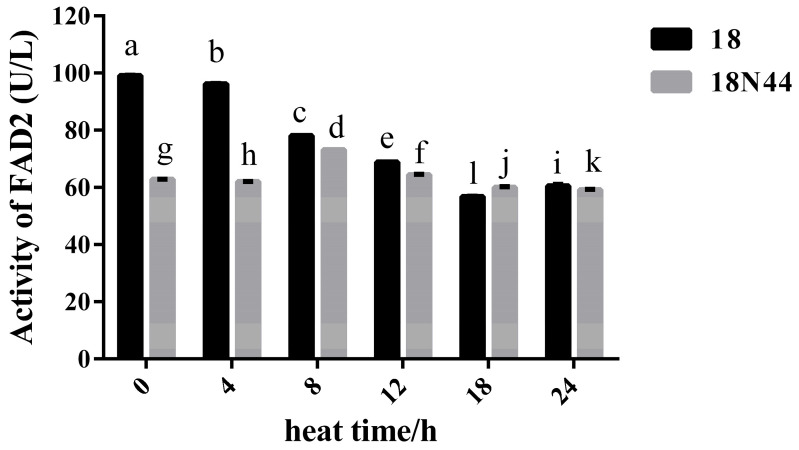
FAD2 activity in mycelia of strains 18 and 18N44. The data are the means of four independent replicates. The statistical analysis was performed using Student’s *t* test, and GraphPad Prism 6 was used to construct the histogram. Bars with different lowercase letters are significantly different at the level of 0.05 (*p* < 0.05).

The enzyme activity of FAD2 in strain 18N44 was lower than that in strain 18. With prolonged heat stress, the activity of FAD2 in strain 18 significantly decreased, while that in strain 18N44 did not significantly change, which may be one of the reasons why strain 18N44 was more resistant to heat stress than strain 18 (Figure 5).

### 3.5. Analysis of the Linoleic Acid Contents of 18 and 18N44 under Heat Stress

**Figure 6:**
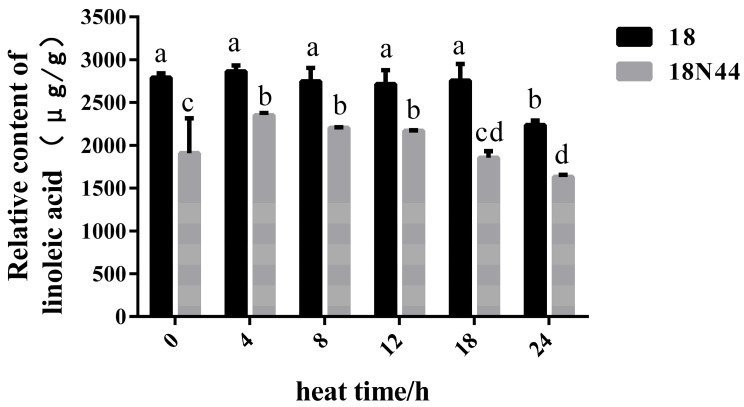
Linoleic acid contents in mycelia of heat-stressed strain 18 and the mutagenic heat-tolerant strain 18N44. Bars with different lowercase letters are significantly different at the level of 0.05 (*p* < 0.05).

Linoleic acid was the main UFA in *L. edodes* strain 18 and the mutagenic heat-tolerant strain 18N44 [23,24]. Gas chromatography-mass spectrometry (GC-MS) was used to determine the linoleic acid content in both strains under heat stress (0, 4, 8, 12, 18, and 24 h). The linoleic acid content decreased significantly after 24 h of heat stress and was significantly greater in strain 18 than in strain 18N44 during heat stress (Figure 6).

## 4. Discussion

FAD2s are widely found in various living organisms, including higher plants, green algae, fungi, animals, and other eukaryotes, as well as prokaryotes such as bacteria. Six FAD2 family candidate genes were obtained by comparing the protein sequences of *L. edodes* in this study. According to the phylogenetic tree construction and protein physicochemical property analysis, the six FAD2 family members were divided into two large clusters. The distribution of introns was generally consistent with the results of phylogenetic tree classification. Great differences exist in the physicochemical properties of the FAD2 protein family members, including differences in amino acid number, isoelectric points, molecular weight, stability, hydrophilicity, and transmembrane domain. However, they all have highly conserved amino acid regions, mainly histidine-enriched segments. Transmembrane domains, histidine motifs, and ER retrieval motifs are commonly found in FAD2 proteins and are considered typical features of FADs [43]. Multiple alignment of six homologous protein sequences from *L. edodes* indicated that they had three regions with strongly conserved histidine motifs (HXXXH, HXXHH, and HXXHH), which had a total of eight histidine residues. These histidine residues are essential for enzymatic catalytic activities and are inferred to act as ligands for the iron atoms in homolog stearoyl CoA desaturase in oilseeds [44]. Mutations at any site of the motif result in the failure of enzyme–endoplasmic reticulum (ER) coupling and attachment to the plasma membrane. The three histidine motifs, located in the N-terminal region (active center of the enzyme), are critical for enzymatic functioning. Their localization requires hydrophobic residues, and amino acid substitution results in the loss of desaturase activity [45]. However, for the homologous FAD2 proteins from *L. edodes*, FAD2-3 and FAD2-5 have no transmembrane helices. These results may indicate consistency among members of the same subfamily and great variation among different subfamilies during the evolution of the *L. edodes* FAD2 family. Many studies have described the important role of the *FAD2* gene in plants [46]. However, the function of FAD2 proteins during fungal responses to abiotic stress has rarely been reported.

The *FAD* gene encodes a key enzyme involved in the production of polyunsaturated fatty acids, which play an important role in plant cold resistance [47,48], but studies on the regulation of the expression of this gene under high-temperature stress have rarely been reported. Zhang et al. reported that the expression of *FAD3* and *FAD8* reduced the resistance of tobacco to high-temperature stress [49]. Silencing of the *FAD7* gene in tobacco plants can increase the ability of the plants to acclimate to relatively high temperatures [50]. Li et al. reported that *CtFAD2-1*, *CtFAD2-2*, and *CtFAD6* were significantly induced in young leaves under cold and heat stress and *CtFAD2-2* and *CtFAD6* were slightly induced in young stems of safflower [51]. *Saccharomyces cerevisiae* cells transformed with the *FAD2-1* gene of sunflowers exhibited the highest percentage of dienoic acids at 10 °C, and the percentage of dienoic acids decreased at higher temperatures [52]. The expression of *Caenorhabditis elegans* Δ12 fatty acid desaturase in *Saccharomyces cerevisiae* cells resulted in the accumulation of 16:2 and 18:2 (linoleic) acids and a growth rate advantage for cells at 12 °C [53]. More studies on *Saccharomyces cerevisiae* and *Rhodotorula toruloides* have indicated that the fatty acid desaturase and *FAD* genes are involved in the stress response to temperature [54,55]. In addition, Sakai et al. studied *Le-FAD1* and *Le-FAD2* in *L. edodes* and reported that the transcription levels of *Le-FAD1* mRNA in the primordium and fruiting body of *L. edodes* were greater than those in mycelia cultivated at 18 °C or 25 °C [56]. In addition, a reduction in growth temperature from 25 °C to 18 °C had no effect on the transcription level of *Le-FAD2* [22]. However, further research on the expression of *Le-FAD1* and *Le-FAD2* under heat stress has not been conducted. In our research, the expression of six homologous *FAD2* genes after heat stress at 37 °C was investigated by RT-qPCR. The results showed that all six *FAD2* genes participated in the heat stress response in *L. edodes*. In particular, the transcription levels of the *FAD2-2*, *FAD2-3*, *FAD2-5*, and *FAD2-6* genes in strains 18 and 18N44 were lower than those in the control sample during heat stress. The transcription levels of *FAD2-2* tended to first decrease and then increase in both strain 18 and strain 18N44, with the lowest values occurring at 12 h and 8 h, respectively, indicating that the *FAD2-2* gene in strain 18N44 responded to heat stress earlier than that in strain 18. The transcription levels of *FAD2-3* in strains 18 and 18N44 continued to decrease with prolonged heat stress time (except for 12 h in strain 18 and 24 h in strain 18N44), and the degree of decline in strain 18N44 was smaller than that in strain 18, indicating that the *FAD2-3* gene in strain 18N44 was less affected by heat stress than that in strain 18. The transcription level of *FAD2-5* fluctuated greatly in strain 18, while the transcription level of *FAD2-5* first decreased then increased, and finally decreased again in strain 18N44 (the inflection points occurred at 8 h and 18 h, respectively). The transcription level of *FAD2-6* in strain 18 first decreased and then increased (the lowest value appeared at 12 h), while it fluctuated significantly in strain 18N44. During heat stress, the transcription levels of *FAD2-2* and *FAD2-3* in strain 18N44 were generally lower than those in strain 18, while the transcription levels of *FAD2-5* in strain 18N44 were greater than those in strain 18. In general, *FAD2-2* and *FAD2-3* may play major roles in strains 18 and 18N44 under heat stress.

Studies have shown that changes in FAD activity are closely related to the plant response to heat stress, and FAD is involved in regulating the fluidity of the cell membrane and reducing heat stress damage by catalyzing the desaturation of fatty acids; namely, a double bond is introduced into fatty acid chains, thus increasing the proportion of unsaturated fatty acids [57]. FAD2, an enzyme closely related to oleic acid 12-hydroxylase, which hydroxylates oleic acid to ricinoleic acid, catalyzes the formation of linoleic acid (18:2) from oleic acid (18:1) [58,59]. Our research showed that the enzyme activity of FAD2 decreased in strain 18 after heat stress but did not significantly change in strain 18N44, and the enzyme activity of strain 18N44 was lower than that of strain 18, which was consistent with the changes in the transcription levels of the *FAD2-2* and *FAD2-3* genes. This may be because the proportion of unsaturated fatty acids was reduced in strain 18 by reducing FAD2 activity after heat stress, thus reducing the damage caused by heat stress. Owing to the greater thermostability of strain 18N44, which did not significantly change after heat stress, strain 18N44 showed lower activity than strain 18, suggesting that high temperature had a greater effect on the heat-sensitive strain 18.

Membrane adaptation to temperature shifts highly depends on adjusting the saturation of fatty acids in membrane lipids [60,61,62,63]. At high growth temperatures, the primary change was the downregulation of linoleic acid and the upregulation of SFAs [64]. After heat stress, PUFA (C18:3) levels in wheat flag leaves decrease during grouting, while the amounts of hexadecenoic acid (C16:1) and linoleic acid (C18:2) increase [65]. These results indicate that the lipid composition of wheat cell membranes changes under thermal stress, which may be an adaptive plant response to heat stress. FA composition analysis of *Histoplasma capsulatum* mycelia suggested that a temperature-tolerant strain (G217B) had a greater SFA concentration and a higher SFA/UFA ratio than did a temperature-sensitive strain [66]. Studies in soybean, Arabidopsis, and other plants have also yielded similar results [67,68,69]. In this study, GC-MS was used to analyze the linoleic acid content of heat-stressed mycelia of strain 18 and the mutagenic heat-tolerant strain 18N44. The 18N44 strain is more tolerant to heat stress, and its linoleic acid content is lower than that of heat-sensitive strain 18. Many reports indicate that the UFA content is positively correlated with cold resistance [70,71,72] and that *FAD2* genes are directly involved in membrane adaptation to temperature stress [73]. This explains the critical role of FAD in regulating membrane fluidity by regulating the degree of fatty acid unsaturation, thereby enhancing resistance to physiological stresses. Our results showed that the transcription levels of the *FAD2-2* and *FAD2-3* genes and the enzyme activity of FAD2 decreased significantly in strain 18 after heat stress, and a decrease in the linoleic acid content was simultaneously detected, which is similar to the results mentioned above.

## 5. Conclusions

FAD2 proteins from *L. edodes* contain three conserved histidine-rich regions (HXXXH, HXXHH, and HXXHH), which include eight histidine residues. Six homologous FAD2 polypeptide sequences were studied using MEGA 7.0 to elucidate the phylogenetic relationships. The distribution of introns was basically consistent with the phylogenetic tree classification results. Based on BLAST results, FAD2-4 and FAD2-6 were in the same clade with a 100% bootstrap value. The mutagenic heat-tolerant strain 18N44 had a lower linoleic acid content and lower FAD2 enzyme activity than wild-type strain 18, and the expression levels of the *FAD2-2* and *FAD2-3* genes under heat stress were significantly lower in strain 18N44 than in strain 18, indicating that FAD2-2 and FAD2-3 may play major roles in the synthesis of linoleic acid during heat stress. When *L. edodes* are subjected to heat stress, FAD2-2 and FAD2-3 may regulate gene expression levels to reduce the linoleic acid content for improved adaptation to heat stress. These data provide an essential basis for the in-depth study of unsaturated fatty acids, especially linoleic acid, and related proteins in *L. edodes* under heat stress and provide theoretical guidance for the breeding of new varieties of *L. edodes* at high temperatures.

## Figures and Tables

**Table 1:** Primer sequences for RT-qPCR.

Gene	Forward Sequence (5′-3′)	Reverse Sequence (5′-3′)
*FAD2-2*	TGCATTTAGGTCGTCGCTGT	GTTGCGAAGAGACCAGTCCA
*FAD2-3*	TCCCATTTATACCGCTGCCC	GACGACGGACTGAAGTGGTT
*FAD2-4*	TGTATGCAGGTGTTGGGCTT	CCTGATCGCTCTTGAGGCTT
*FAD2-5*	TCAAGAGCCACGGATGGAAC	AATCGTCGGATCGCTGTGTT
*FAD2-6*	CAAAACTCGCTTCGCCACAA	TTGGAAGGCATAAACGGGGT
*FAD2-8*	ATGCCTTTCTACCACGGTCC	ACACAACATCACCCTCGTCC
*TUB*	GACATTTGCTTCCGAACCCT	CGGACATAACAAGGGACACA

**Table 2:** Physicochemical properties of homologous FAD2 proteins from *L. edodes*.

Protein	Amino Acids	MW (Da)	PI	Atomic Formula	Instability Index	GRAVY	(Arg + Lys) Residues	(Asp + Glu) Residues
FAD2-2	361	40,169.33	6.61	C_1864_H_2805_N_485_O_490_S_10_	37.92	0.219	22	26
FAD2-3	421	48,311.55	8.74	C_2222_H_3340_N_586_O_594_S_17_	33.39	−0.105	40	34
FAD2-4	413	47,651.61	8.93	C_2200_H_3291_N_575_O_590_S_13_	41.30	−0.015	39	32
FAD2-5	427	49,620.25	8.92	C_2314_H_3449_N_587_O_605_S_14_	37.87	−0.076	44	38
FAD2-6	416	48,173.29	9.22	C_2238_H_3314_N_578_O_588_S_14_	44.70	0.003	38	29
FAD2-8	422	49,625.57	6.39	C_2328_H_3351_N_587_O_603_S_13_	30.20	−0.287	41	47

## Data Availability

The dataset supporting the conclusions of this study is available, and the authors have agreed to share the dataset. Readers can contact the authors by email to obtain the raw data used in this manuscript.

## Supplementary Materials

The following supporting information can be downloaded at: https://www.mdpi.com/article/10.3390/jof10070496/s1, Figure S1: Prediction of N-terminal signal peptides in homologous FAD2 proteins from *L. edodes*. Table S1: Prediction of the subcellular locations of homologous FAD2 proteins from *L. edodes*.
