# Supplementary material for: Homologous Delta-12 Fatty Acid Desaturase (FAD2) Genes Affect Gene Expression and Linoleic Acid Levels in Lentinula edodes under Heat Stress
Source: J Fungi (Basel). 2024 Jul 18;10(7):496. doi: 10.3390/jof10070496 (PMC11277945; doi:10.3390/jof10070496)
Supplement: Supplementary file 1 [file jof-10-00496-s001.zip › jof-3046907-supplementary.pdf]

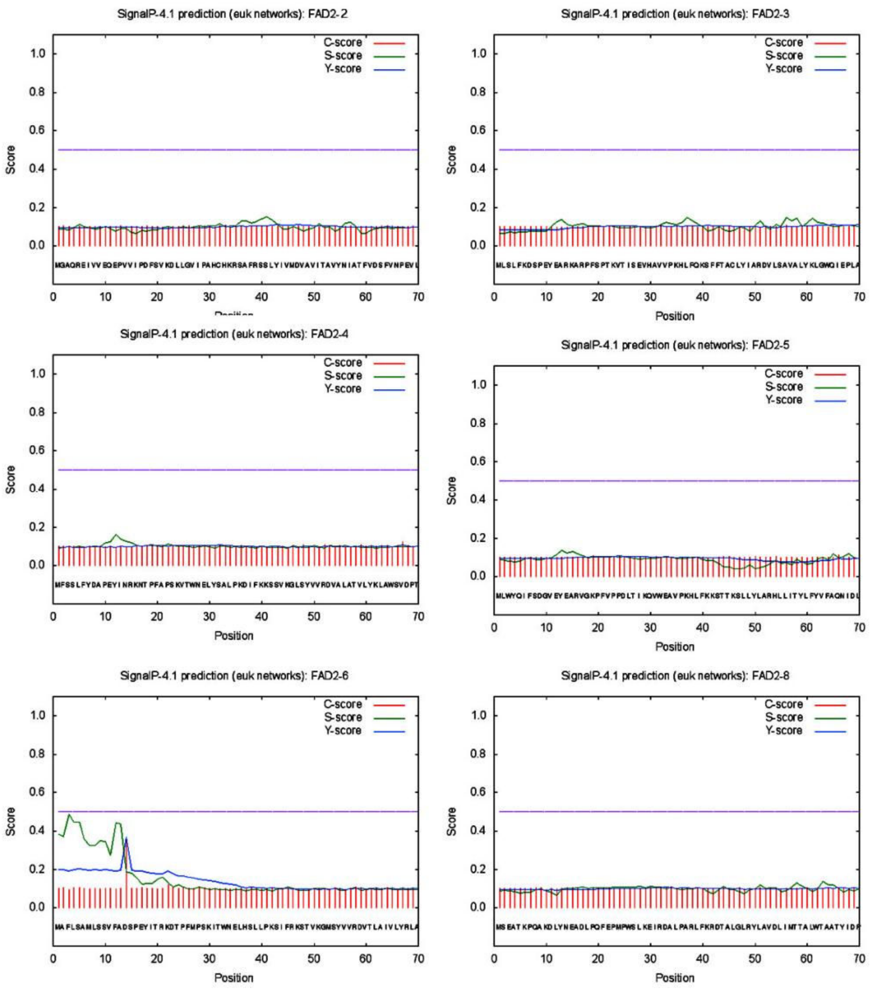

Fig. S1. Prediction of N-terminal signal peptides in homologous FAD2 proteins from *L. edodes.* Occurrence of signal peptide cleavage sites was predicted by SignalP4.1: C denotes raw cleavage site score, S denotes signal peptide score and Y denotes combined cleavage site score.

Table S1. Prediction of the subcellular locations of homologous FAD2 proteins from *L. edodes*

Name	Length	mTP	SP	other	Loc	RC
FAD2-2	361	0.120	0.094	0.848	-	2
FAD2-3	421	0.561	0.075	0.377	M	5
FAD2-4	413	0.263	0.116	0.678	-	3
FAD2-5	427	0.119	0.249	0.731	-	3
FAD2-6	416	0.247	0.656	0.274	S	4
FAD2-8	422	0.084	0.116	0.903	-	2

Subcellular localizations of *L. edodes* FAD2 proteins were predicted by TargetP 1.1. N-terminal presequences predicted by a mitochondrial targeting peptide (mTP) or a secretory pathway signal peptide (SP) were referred to assign location. Prediction of localization (Loc), Mitochondrion (M), the sequence contains mTP, a mitochondrial targeting peptide; Secretory pathway (S), the sequence contains SP, a signal peptide. Reliability class (RC), from 1 to 5, where 1 indicates the strongest prediction.
